# Pathways and progress to enhanced global sexually transmitted infection surveillance

**DOI:** 10.1371/journal.pmed.1002328

**Published:** 2017-06-27

**Authors:** Melanie M. Taylor, Eline Korenromp, Teodora Wi

**Affiliations:** 1 Department of Reproductive Health and Research, World Health Organization, Geneva, Switzerland; 2 Division of STD Prevention, Centers for Disease Control and Prevention, Atlanta, Georgia, United States of America; 3 Avenir Health, Geneva, Switzerland

## Abstract

Melanie Taylor and colleagues discuss global initiatives for surveillance of sexually transmitted diseases.

**Summary points**

- WHO recommends 4 components of sexually transmitted infection (STI) surveillance at the country level: (1) clinical case reporting, (2) infection prevalence surveys, (3) assessment of the etiologies of STI syndromes, and (4) monitoring of antimicrobial resistance.
- The WHO Global Health Sector Strategy on STI includes the following targets for achievement by 2030: (1) a 90% reduction of syphilis incidence, (2) a 90% reduction in gonorrhoea incidence, and (3) 50 or fewer cases of congenital syphilis per 100,000 live births in 80% of countries.
- WHO’s global STI reporting and estimation currently utilizes 3 systems: (1) Global AIDS Response Progress Reporting System (GARPR) (recently renamed Global AIDS Monitoring), (2) the Spectrum-STI estimation tool, and (3) Gonococcal Antimicrobial Surveillance Program (GASP).
- Surveillance data reported through these systems can be used to monitor and enhance prevention interventions and guide resources to improve STI services and reduce STI morbidity.
- WHO offers technical support to countries interested in the following: (1) improving STI data collection and reporting through GARPR, (2) expanding laboratory capacity through GASP, and (3) generating national estimations using the Spectrum-STI tool.
- Enhancing STI surveillance through expanded use of these systems is needed within the context of reducing global STI burden and monitoring goals and indicators within the WHO Global Health Sector Strategy on Sexually Transmitted Infections (2016–2021).

## Introduction

Sexually transmitted infections (STIs) are the most prevalent communicable diseases worldwide. WHO estimated that over 350 million curable STIs occurred globally in 2012, corresponding to nearly 1 million new cases occurring each day [1]. Viral STIs pose an additional burden, with estimates of more than 400 million prevalent cases of herpes simplex and nearly 300 million women infected with human papillomavirus [2–3]. In comparison, WHO reported an estimated 36.7 million people globally living with HIV infection in 2015 [4] and an estimated 240 million people with chronic hepatitis B [5]. Though largely preventable, these STIs continue to cause significant morbidity and mortality. Surveillance of STIs remains a critical component of global monitoring and response.

In 2016, the 69th World Health Assembly adopted 3 integrated global health sector strategies on HIV, viral hepatitis, and STIs for the period 2016–2021 [6]. Each defines global targets dependent on strong disease surveillance systems for progress monitoring. The following are included in the WHO Global Health Sector Strategy on STIs (2016–2021) as global targets for achievement by 2030: (1) a 90% reduction in syphilis incidence, (2) a 90% reduction in gonorrhoea incidence, and (3) 50 or fewer cases of congenital syphilis per 100,000 live births in 80% of countries [2]. Historically, STI surveillance data have been poor and flawed by underreporting, unreliability, lack of representation, limited geographic scope, and changing methodologies. Monitoring of progress toward the global targets requires improved monitoring systems at national, regional, and global levels, which routinely include timely, consistent, and accurate incidence or prevalence data. Reliable surveillance information also sets the foundation for program evaluation and optimization or course adjustment in prevention and treatment strategies and for advocacy, strategic planning, and resource mobilization.

WHO recommends standard indicators and methodologies by which countries can gather surveillance data to produce reliable STI data to guide program activities [7–9]. Specifically, WHO identifies 4 components of STI surveillance: (1) clinical case reporting, (2) infection prevalence surveys, (3) assessment of the etiology of STI syndromes, and (4) monitoring of antimicrobial resistance. These data should then be used to track the epidemiology of STIs, monitor and improve prevention interventions, and inform treatment guidance with the ultimate goal of tracking and reducing STI morbidity and mortality [9]. Currently, most country-level STI surveillance is based on monitoring of clinical cases of common STI syndromes, such as urethral discharge and genital ulcer disease. It is recommended that national case reporting should include syphilis, gonorrhoea, urethral discharge, and genital ulcer disease, ideally with data disaggregated by gender and age. Among countries using STI syndromic case management and surveillance, etiologic surveys should be performed periodically to guide treatment recommendations [2]. Special-focus populations such as pregnant women, men who have sex with men, sex workers, adolescents, and sexual partners of index cases should be included in routine syphilis prevalence screening [7–9]. WHO offers assistance to countries to improve STI surveillance using these key components.

WHO’s global STI reporting and estimation currently utilizes 3 systems. The first is the Global AIDS Response Progress Reporting System (GARPR), recently renamed Global AIDS Monitoring, wherein countries are asked to report annually on syphilis prevalence in various populations and on case rates for syphilis, gonorrhoea, and urethral discharge [10]. The second is the Spectrum-STI estimation tool, developed in 2016 with Avenir Health, which countries can use to collate and review national STI data and estimate prevalence and incidence of STIs in their national adult population, including historic and ongoing time trends [11]. This tool uses as its primary inputs the STI indicator data reported through GARPR. The third system is the Gonococcal Antimicrobial Surveillance Program (GASP). WHO, in collaboration with reference labs in 60 countries, performs laboratory surveillance on the emergence of antimicrobial resistance among gonococcal isolates [12]. This paper describes each of these systems in turn, along with opportunities to enhance their utility for improving programs.

## GARPR

Since 2014, WHO has partnered with the Joint United Nations Programme on HIV/AIDS (UNAIDS) to include selected STI coverage and impact indicators in GARPR [10]. GARPR collates country-reported HIV/AIDS outcome and coverage indicators used to monitor progress made towards national and global HIV targets [13–14] STI indicators were incorporated into GARPR in 2011 (inclusive of data from 2008–2011) and first reported in 2012 [10], focusing on syphilis screening and treatment in antenatal care (ANC), the number of new cases of gonorrhoea, STI syndrome cases (urethral discharge and genital ulcer disease) and prevalence of syphilis among key populations (S1 Table) [7,9,12,15–17]. GARPR also includes questions regarding the availability of a national STI prevention plan and syphilis test type.

The WHO STI surveillance report summarizes trends in STIs globally based on GARPR-reported data [12]. For syphilis screening indicators in ANC, in the 2013–2014 GARPR reporting period, the African region had the largest number of reporting countries for all syphilis ANC indicators (S2 Table). Overall, median syphilis screening coverage in ANC ranged from a high of 100% in the Western Pacific region to 40% in the African region. GARPR-reported median treatment coverages were higher at 96% (range: 6.1%–100%), but these data came from fewer countries and may not be representative. Rates of urethral discharge and gonorrhoea cases among men were reported by 56 and 53 countries, respectively. The rate of urethral discharge cases was highest in the African region at 568.6 per 100,000 adult males, while the rate of gonorrhoea cases was highest in the Western Pacific region at 88.6 per 100,000 adult males (Table 1). Despite this wide variation, case reporting rates in most countries are much below the case rates that would be expected based on population-based prevalence and incidence estimates [1]. This reflects that only a fraction of infection episodes are symptomatic, only a fraction of symptomatic episodes are treated through qualified health services, and only a fraction of symptomatic episodes treated through qualified health services are recorded with the notification transferred through the national reporting system.

**Table 1:** Male urethral discharge (UD) rate and male gonorrhoea case reporting rates by region, 2014.

WHO region	Countries in region	Countries reporting male UD	Median male UD case rate* (range)	Countries reporting male gonorrhoea	Median male gonorrhoea case rate* (range)
African region	47	20	568.6 (30.1–3,579)	5	50.1 (7.2–238)
Region of the Americas	35	11	74.6 (10.0–400)	18	29.3 (1.9–153)
Eastern Mediterranean region	21	8	24.1 (7.3–614)	6	3.2 (0.9–385)
European region	53	1	223	9	25.5 (2.9–61)
Southeast Asia region	11	5	121 (5.0–314)	4	7.0 (2.4–20)
Western Pacific region	27	11	141 (9.0–1,171)	11	88.6 (0.5–317)
All regions	194	56	144 (5.0–3,579)	53	25.5 (0.5–386)

* UD and GC case rate: per 100,000 men aged 15–49 years. Source: WHO Global STI Surveillance report 2015 [9].

## Future directions in STI surveillance using GARPR

WHO and UNAIDS collect and share the GARPR-reported country indicator data in a regional and global context through the Global Health Observatory (GHO) [17]. Countries that enter data into GARPR through the GHO can create interactive maps and charts. STI program and reporting improvements can be seen and tracked in GARPR by country and region. Indicators recommended for GARPR reporting are aligned with indicators proposed by WHO for monitoring and evaluating progress and impact of the STI Global Health Sector Strategy [2], for which the Spectrum-STI estimation tool can be used to estimate baseline burdens and the burden reductions and impact over time.

## Spectrum-STI estimation tool

The Spectrum-STI estimation model was built in the Spectrum suite of health policy models in 2016 to enable country-owned estimations of STI prevalence and incidence in national adult populations. Spectrum includes modules that support estimation of national burdens, trends, service needs and program impact for family planning, HIV/AIDS [18–20], and other health areas, building on WHO-recommended standardized disease surveillance indicators that most national disease programs routinely collect. For HIV/AIDS and STIs, the burden indicators used align with those collected through the GARPR system. As of 2016, the STI estimation covered gonorrhoea and syphilis, the 2 STIs for which the 2016–2021 WHO Global Health Sector Strategy for STI control set impact goals (i.e., to reduce, from 2018 to 2030, the number of incident cases of gonorrhoea and syphilis by 90%) [2] and for which GARPR has included dedicated indicators (S2 Table), as well as chlamydia.

Data requirements, statistical estimation methods, and assumptions used by this STI trend estimation model are described elsewhere [11]. In brief, for syphilis the estimation is anchored on ANC prevalence data collected through routine programmatic screening and periodic sentinel surveys, supplemented where available with any general population surveys. These surveillance data are then fitted through a statistical regression model that weighs each data point according to its national coverage and representativeness [11]. For gonorrhoea and chlamydia, where fewer representative population surveys are available, prevalence data from the country and the geographical region of which the country is part can be used. A “moving-average” statistical model combines all qualifying data, applying highest statistical weight to data from the country itself, if available [11]. In this statistical model, these prevalence data can be complemented with gonorrhoea and/or UD case reports (in those countries where the case reporting time series is judged to be of sufficient and stable completeness and quality) to inform the time trend (S1 Fig).

The Spectrum-STI module, which is freely available online [21], is preloaded with country data reported through GARPR for more than 150 countries, plus any prevalence survey data that were included in WHO’s 2005, 2008, and 2012 global and regional estimates [1] and other systematic literature reviews and meta-analyses. This allows for an immediate, easy, and quick start to an estimation, in which the user can update, complete, and correct the data and assign statistical weights according to the latest in-country evidence and insights on data quality and representativeness.

Use of this standardized epidemiological modelling framework offers countries the option to review and synthesize STI surveillance indicator data and to use the resulting STI trend estimates to inform STI program planning and evaluation [11]. As of May 2017, Spectrum-STI estimation has been applied by national STI program staff and in-country STI experts and stakeholders in Zimbabwe, Morocco, Mongolia, and Colombia. In Morocco, 2016 estimates for gonorrhea, chlamydia and syphilis were agreed upon among stakeholders, and the associated burden of UD resulting from gonorrhoea and chlamydia assessed to gauge the completeness of clinical UD case reporting [11,22]. The chlamydia and UD reporting completeness data are not yet part of the publicly available user interface software. The STI estimates in Morocco informed the country’s new HIV/STI strategy for 2017–2021 (S3 Table). Across countries that have applied the tool so far, Spectrum-supported STI estimations have illustrated the limited prevalence data for gonorrhoea and chlamydia. GARPR-reported gonorrhoea and/or UD case reports are an important input to estimating gonorrhoea prevalence trends, but robust estimation of prevalence and incidence levels requires at least 1 recent population-based prevalence survey. For syphilis, the data base for estimations is more solid; over 150 countries have recent prevalence data from either periodic ANC sentinel surveys and/or routine programmatic ANC screening, most of which is shared publicly through GARPR. Continued data improvements are expected as many countries have transitioned or are successfully transitioning from periodic ANC sentinel surveys to continuous routine monitoring of programmatic ANC syphilis screening, of which the coverage is increasing.

## Future directions in STI surveillance using Spectrum

Up to 8 country applications of Spectrum-STI are foreseen in 2017 as a start to a multiyear global rollout plan. The long-term vision is inspired by the successful earlier rollout of the Spectrum HIV estimation module, which since its creation in 2002 is used by over 150 countries for annual HIV burden estimations, with UNAIDS support and biannual regional training exercises. For syphilis, a multicountry estimation of adult prevalence is underway in 2017 using GARPR-reported data to underpin the WHO’s next (2018) global and regional STI burden estimation and extend that with a first-ever historic regional-level time trend estimation. Building on the global syphilis estimates for women in ANC, Spectrum results are now furthermore being used to estimate trends in congenital syphilis, its drivers, and the impact of ANC-based screening and treatment services in preventing congenital syphilis. In February 2017, a pilot was completed in Mongolia, during which the WHO’s congenital syphilis estimation tool [23–24] was integrated into the Spectrum-STI module.

## Gonorrhoea antimicrobial resistance monitoring

Gonorrhoea is one of the most common bacterial STIs worldwide [1]. The emergence of antimicrobial resistance among *Neisseria gonorrhoeae* isolates is a threat to control of this STI. Surveillance for the spread of resistance is critical, as few options remain for treatment beyond current cephalosporin-based regimens. GASP leads monitoring of the susceptibility patterns of gonococcal isolates. Using a collaboration of laboratories in more than 60 countries in 6 regions, GASP is positioned to monitor gonococcal antimicrobial resistance globally and informs treatment recommendations [12]. Currently, the WHO European region accounts for the majority of GASP-reporting countries, while the African and Eastern Mediterranean regions have the fewest reporting countries. Participation in GASP requires laboratory infrastructure sufficient for etiologic diagnosis of gonorrhoea as well as capacity to perform antimicrobial susceptibility testing. Many high-morbidity countries rely on syndromic management of STIs and lack capacity for etiologic testing. Advocacy for improving country-level capacity to perform routine or periodic etiologic diagnosis and gonococcal antimicrobial susceptibility monitoring is needed, particularly among high-morbidity countries.

Since 2009, 46 countries have reported gonococcal isolates with reduced susceptibility to currently recommended cephalosporins. In 2012–2013, 4 countries in the Western Pacific reported reduced cephalosporin susceptibility, defined as elevated minimum inhibitory concentrations of cefixime (0.25 ug/ml) or ceftriaxone (>0.125 ug/ml) in >5% of gonococcal isolates, the established cutoff for changing treatment guidelines. Only 1% of gonococcal isolates reported from the European region demonstrated decreased sensitivity to ceftriaxone; however, 9 countries in this region reported reduced susceptibility to cefixime in 5% or more of isolates. Seventeen of 42 countries reported resistance to azithromycin among >5% of isolates (Table 2). Quinolone susceptibility data was reported by 56 countries; a majority of these reported quinolone resistance among >20% of gonococcal isolates. Quinolone resistance among greater than 90% of gonococcal isolates was reported by 12 countries, mainly in Asia.

**Table 2:** Gonococcal reduced susceptibility to cephalosporins and resistance to azithromycin or quinolones, as reported by countries and regions through the 2012–2013 round of GASP.

Antibiotic class and select regions with GASP reporting	Number of countries reporting susceptibility testing	Number of countries reporting reduced cephalosporin susceptibility* in ≥5% of samples or azithromycin or quinolone resistance	Percent of reporting countries with ≥5% reduced cephalosporin susceptibility or azithromycin or quinolone resistance
Cephalosporins	49	29	59%
Western Pacific	14	4	29%
European	24	9	38%
Southeast Asia*	7	5	71%
Azithromycin	42	17	40%
European	24	13	54%
Western Pacific	14	1	7.1%
Quinolones	56	55	98%
Americas	11	10	91%
European	24	24	100%
Southeast Asia	5	5	100%
Western Pacific	14	14	100%

*Elevated minimum inhibitory concentrations of cefixime (0.25 ug/ml) or ceftriaxone (>0.125 ug/ml).

**Reporting aggregated over (any year or years within) 2009–2013. Source: WHO Global STI Surveillance report 2015 [9].

## Future directions in AMR surveillance through GASP

Efforts to improve and expand GASP laboratory reporting sites are recommended by WHO but are challenged by limited national advocacy, prioritization, and funding for many countries. Data are limited even from GASP-reporting countries, which hampers interpretation in terms of time trends and geographical variations in resistance levels [12]. Increased efforts are needed to expand gonococcal isolate testing within GASP-reporting countries to increase representation and improve surveillance for reduced cephalosporin susceptibility. Establishment of gonococcal antimicrobial resistance monitoring is needed, particularly within countries and regions with high gonorrhoea burden. Results from GASP demonstrate widespread gonococcal isolate resistance to azithromycin and quinolones and emergence of resistance to cephalosporins, with data collected from less than half of countries.

## Conclusions

International support to standardize STI reporting through GARPR and GASP and use of the Spectrum estimation tool have improved STI surveillance in countries and at a global level. WHO offers technical support to countries interested in improving STI data collection and reporting through GARPR and expanding laboratory capacity through GASP, as well as support for national estimations using the Spectrum-STI tool (S4 Table).

Challenges in enhancing STI surveillance are numerous. Funding for STI surveillance falls far short of what is needed, and STI surveillance is often the weakest within countries and regions with the highest burdens [25]. New systems such as those described here must still address incomplete reporting, poor STI and laboratory surveillance infrastructure, and the limitations of estimating STI prevalence and incidence where testing and case data are lacking. Access to sensitive and specific diagnostics for other STIs are limited in low- and middle-income countries, resulting in limited data sources for surveillance. UNAIDS limits the number of STI indicators that are allowed in the GARPR system because of the reporting burden on countries; therefore, only STIs monitored in the global strategy are included (syphilis and gonorrhoea). However, the Spectrum-STI tool can be used to estimate rates and trends of syphilis, gonorrhea and chlamydia, provided that appropriate data are available. Other health areas, notably HIV/AIDS, provide a good example of how strong sustained national commitment and international support, both financial and technical, can improve technical capacity, surveillance processes and systems in countries, and result in increased quality of data, estimates, and strategic program planning.

Improvements in STI surveillance depend on opportunities to secure funding, implement new technologies and tools, and link with other systems [25]. For example, the use of rapid dual HIV/syphilis test kits should enable expansion of routine syphilis screening in priority populations such as pregnant women [26]. Adolescent HPV screening and vaccination programs may offer the opportunity to provide chlamydia and gonorrhoea screening [2]. Spectrum-STI–based national burden estimations may be most feasibly rolled out when integrated with periodic regional or country training exercises conducted periodically for Spectrum-supported country HIV Spectrum estimations. Finally, laboratory capacity strengthening and expansion of the number of laboratories participating in GASP can improve the ability to detect and monitor the emergence of gonococcal antimicrobial resistance.

In summary, STI surveillance merits further strengthening, building on enhanced global guidance from WHO and the GARPR and GASP systems as well as the Spectrum-STI estimation tool. It is critical to expand STI screening, treatment, prevention, and surveillance within evolving health systems, integrating where appropriate with programs such as those for HIV prevention and treatment, family planning, and maternal child health.

## Supporting information

S1 Table STI indicators included in GARPR. (DOCX)

S2 Table Antenatal syphilis screening coverage, prevalence of active syphilis in women in ANC tested (in routine screening or in sentinel surveillance), and syphilis treatment coverage in women in ANC diagnosed during routine ANC screening, as reported by countries into the GARPR system (reporting years 2013–2014). (DOCX)

S3 Table Use of Spectrum-STI estimates to inform Morocco’s HIV/STI strategy for 2017–2021. (DOCX)

S4 Table Country access to and use of GARPR, the Spectrum-STI tool, and GASP. (DOCX)

S1 Fig Structure of the Spectrum-STI estimation model. (TIF)

## Abbreviations

ANC: antenatal care
GARPR: Global AIDS Response Progress Reporting System
GASP: Gonococcal Antimicrobial Surveillance Program
GHO: Global Health Observatory
STI: sexually transmitted infection
UD: urethral discharge
UNAIDS: Joint United Nations Programme on HIV/AIDS
