# Supplementary material for: Pathways and progress to enhanced global sexually transmitted infection surveillance
Source: PLoS Med. 2017 Jun 27;14(6):e1002328. doi: 10.1371/journal.pmed.1002328 (PMC5486957; doi:10.1371/journal.pmed.1002328)
Supplement: S1 Table — (DOCX) [file pmed.1002328.s001.docx]

**S1 Table: STI Indicators included in the Global AIDS Response Progress Reporting System (GARPR)**

| **GARPR Indicator** | **Population** |
| --- | --- |
| Percentage of women accessing antenatal care (ANC) services who were tested for syphilis | Pregnant women |
| Percentage of antenatal care attendees tested who were positive for syphilis; | Pregnant women |
| Percentage of antenatal care attendees positive for syphilis who received treatment; | Pregnant women |
| Rate of reported congenital syphilis cases (live births and stillbirths) in the past 12 months (per 100,000 live births). | Infants |
| Percentage of sex workers with active syphilis | Sex workers |
| Percentage of men who have sex with men with active syphilis | Men who have sex with men |
| Rate of clinically diagnosed (syndromic diagnosis) cases of urethral discharge among men | Men ages 15-49 |
| Rate of male gonorrhoea cases based on laboratory diagnosis | Men ages 15-49 |
| Rate of clinically diagnosed cases of genital ulcer disease among men and women (removed in 2015); | Men and women ages 15-49 |
