# Supplementary material for: Pathways and progress to enhanced global sexually transmitted infection surveillance
Source: PLoS Med. 2017 Jun 27;14(6):e1002328. doi: 10.1371/journal.pmed.1002328 (PMC5486957; doi:10.1371/journal.pmed.1002328)
Supplement: S2 Table — (DOCX) [file pmed.1002328.s002.docx]

**S2 Table: Antenatal syphilis screening coverage, prevalence of active syphilis in ANC women tested (in routine screening, or in sentinel surveillance), and syphilis treatment coverage in ANC women diagnosed during routine ANC screening, as reported by countries into the GARPR system, reporting years 2013-2014***

| WHO Region | Countries in Region | Number of countries reporting syphilis testing of pregnant women during ANC* | Median percent [range] of ANC testing coverage | Number of countries reporting syphilis test positivity among ANC attendees* | Median percent [range] who tested positive | Number of countries reporting treatment of ANC attendees with syphilis* | Median percent [range] who received treatment |
| --- | --- | --- | --- | --- | --- | --- | --- |
| African Region | 47 | 34 | 40 [0.7-100] | 31 | 1.6 [0-11] | 21 | 98 [6.1-100] |
| Region of the Americas | 35 | 19 | 88 [17-100] | 21 | 0.4 [0-3.1] | 19 | 93 [50-100] |
| Eastern Mediterranean Region | 21 | 5 | 43 [6-100] | 4 | 0.0 [0.0-1.5] | 3 | 80 [47-100] |
| European Region | 53 | 9 | 93 [86-100] | 9 | 0.1 [0.0-1.0] | 7 | 100 [29-100] |
| South-East Asia Region | 11 | 7 | 58 [1.2-97] | 7 | 0.5 [0.1-1.7] | 6 | 90 [21-100] |
| Western Pacific Region | 27 | 15 | 100 [45-100] | 13 | 1.8 [0-13.5] | 10 | 100 [64-100] |
| **Overall** | **194** | **89** | **86 [0.7-100]** | **85** | **0.7 [0-13.5]** | **66** | **96 [6.1-100]** |

*Countries that reported in either 2013 or 2014 or in both years; typically data from routine ANC screening is annual, whereas ANC sentinel surveillance, a complementary or alternative source of syphilis prevalence data, is conducted every two years.

Source: WHO Global STI Surveillance report 2015
