# Supplementary material for: Pathways and progress to enhanced global sexually transmitted infection surveillance
Source: PLoS Med. 2017 Jun 27;14(6):e1002328. doi: 10.1371/journal.pmed.1002328 (PMC5486957; doi:10.1371/journal.pmed.1002328)
Supplement: S3 Table — (DOCX) [file pmed.1002328.s003.docx]

**S3 Table: Use of Spectrum STI estimates to inform Morocco’s HIV/STI strategy 2017-2021**

| 1. The Spectrum syphilis estimation urged the national HIV/STI program to kick-start its planned new information system to collect syphilis prevalence data from routine ANC-based screening, to replace the earlier, now discontinued surveillance through periodic ANC sentinel surveys. 2. The estimations in Morocco highlighted a persistently high burden of untreated chlamydia (relative to gonorrhea, which dominates UD cases seen in clinics), which due to a frequent lack of symptoms benefits less from routine, clinic-based services than gonorrhea, and which merits additional community-based screening and treatment approaches. 3. The Morocco estimation suggested historic and ongoing declines in the prevalence of the STIs modelled, which could be partly attributed to improving STI treatment access and coverage, associated with the national introduction of the syndromic approach in 2000; in parallel, the reporting completeness of UD cases was estimated to have increased between 1995 and 2016 [20]. |
| --- |
