# Supplementary material for: Pathways and progress to enhanced global sexually transmitted infection surveillance
Source: PLoS Med. 2017 Jun 27;14(6):e1002328. doi: 10.1371/journal.pmed.1002328 (PMC5486957; doi:10.1371/journal.pmed.1002328)
Supplement: S4 Table — (DOCX) [file pmed.1002328.s004.docx]

**S4 Table: Country Access to and Use of GARPR, Spectrum STI Tool, and GASP**

| **STI Reporting System** | **Joining the system and data entry** | **Data Viewing** |
| --- | --- | --- |
| Global AIDS Response Reporting System (GARPR) | Send email to: [aidsreporting@unaids.org](mailto:aidsreporting@unaids.org) for instructions on reporting | <http://apps.who.int/gho/data/node.main> |
| Spectrum STI Estimation Tool | Available for free download:  http:avenirhealth.org/software-spectrum.php | There is no current systems for country-updated files to be viewed by other users |
| Gonococcal Antimicrobial Surveillance Program (GASP) | Potential users and country programs can indicate their interest via the WHO regional office | WHO STI Surveillance Report [9] |
