# Supplementary figures and images for: Pathways and progress to enhanced global sexually transmitted infection surveillance
Source: PLoS Med. 2017 Jun 27;14(6):e1002328. doi: 10.1371/journal.pmed.1002328 (PMC5486957; doi:10.1371/journal.pmed.1002328)

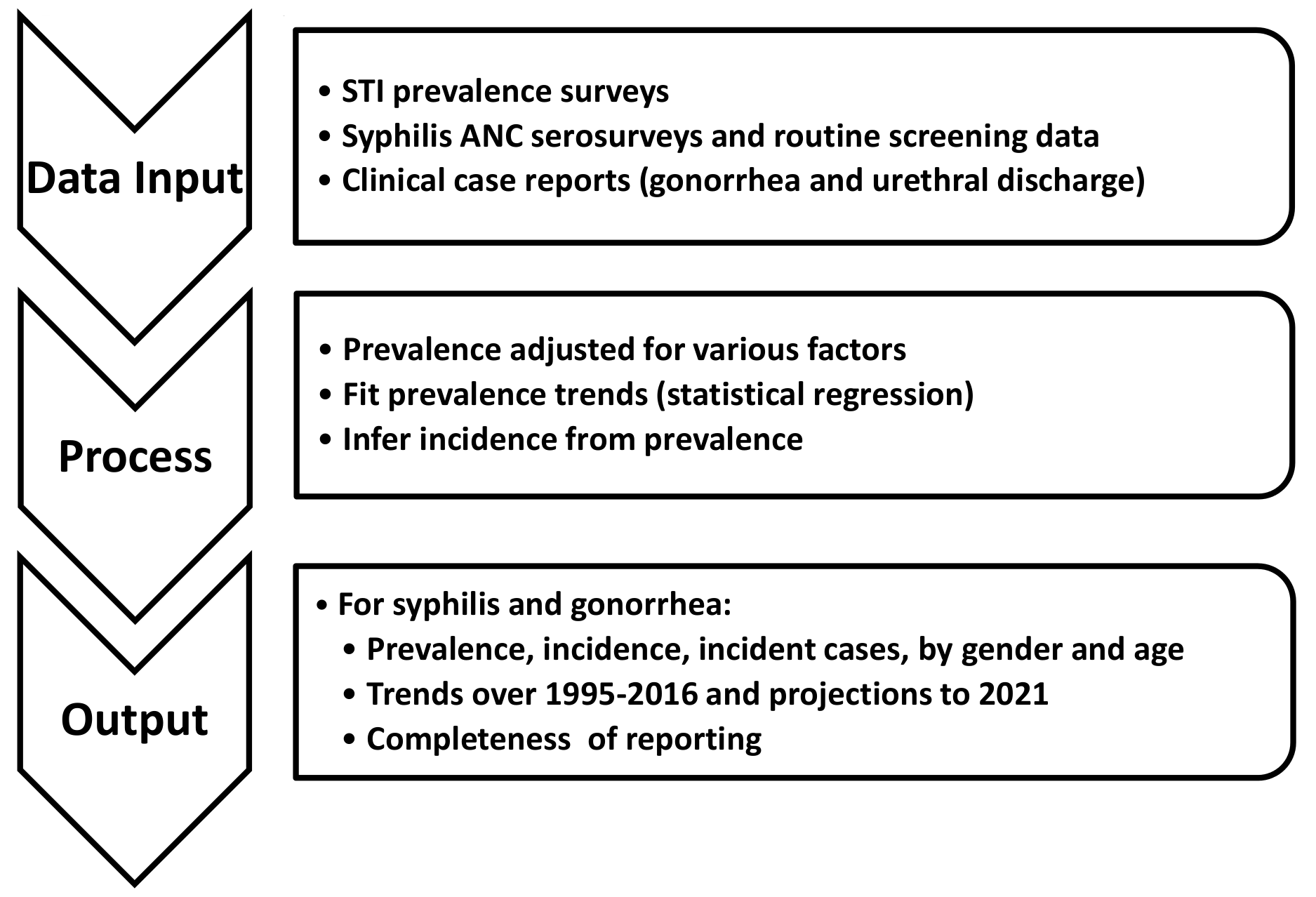

Supplement: S1 Fig — (TIF) [file pmed.1002328.s005.tif]
